# Leptospirosis vaccination in dogs attending UK primary care practices: vaccine uptake and factors associated with administration

**DOI:** 10.1186/s12917-022-03382-6

**Published:** 2022-07-22

**Authors:** Collette Taylor, Dan G. O’Neill, Brian Catchpole, Dave C. Brodbelt

**Affiliations:** Pathobiology and Population Sciences, The Royal Veterinary College, Hawkshead Lane, North Mymms, Hatfield, Herts AL9 7TA UK

**Keywords:** Leptospirosis, Vaccination, Epidemiology, EPR, Electronic patient record, Dog, Primary-care, Risk factors, Incidence, VetCompass

## Abstract

**Background:**

Leptospirosis is a zoonotic disease that is found globally and affects most mammalian species. Vaccination of dogs against leptospirosis is an important approach to preventing clinical disease, or reducing disease severity, as well as reducing transmission of the infection to humans. Although it is generally considered to be a ‘core’ vaccine, there is limited information on the level of leptospirosis vaccine usage and factors associated with its usage in dogs in the UK. The study aimed to report the uptake of leptospirosis vaccination and factors associated with its usage in a cohort of dogs under primary veterinary care during a 12-month period.

**Results:**

From a population of 905,543 dogs, 49% (95%CI 48.9–49.1%) had at least one leptospirosis vaccine administered during the 12 months of study. Adult dogs had reduced odds of receiving a leptospirosis vaccine when compared to dogs < 1 year old, with dogs > 8 years old having a greater than ten-fold reduction in odds (OR = 0.08, 95%CI 0.07–0.09). Odds of receiving a leptospirosis vaccine was increased in insured dogs when compared to uninsured dogs (OR = 1.22, 95%CI = 1.17–1.28). Neutered dogs had reduced odds of receiving a leptospirosis vaccine (OR = 0.87, 95%CI 0.83–0.91). Breed associations with receiving a leptospirosis vaccine varied. Several breeds were associated with increased odds of receiving a leptospirosis vaccine when compared to crossbreed dogs, including Border Terriers (OR = 1.49, 95%CI 1.42–1.57), Golden Retrievers (OR = 1.30, 95%CI = 1.24–1.37), Cocker Spaniels (OR = 1.27, 95%CI 1.23–1.31) and West Highland White Terriers (OR = 1.27, 95%CI 1.22–1.31). French Bulldogs (OR = 0.64, 95%CI = 0.62–0.67), Staffordshire Bull Terriers (OR = 0.79, 95%CI 0.78–0.82) and Pugs (OR = 0.91, 95%CI =0.88–0.95) had significantly reduced odds of receiving a leptospirosis vaccination during the study.

**Conclusion:**

This work identified that almost half of the UK primary care attending population received a leptospirosis vaccine during the year. Several demographic variables were associated with leptospirosis vaccine administration, with age being particularly important. Both the proportion of uptake and factors associated with leptospirosis vaccine usage can be used as a benchmark for comparisons in the future. Additionally, an understanding of which populations have reduced odds of receiving a leptospirosis vaccine can potentially be used for initiatives to encourage owner vaccination uptake in these groups.

**Supplementary Information:**

The online version contains supplementary material available at 10.1186/s12917-022-03382-6.

## Introduction

Leptospirosis is a disease in dogs that can cause substantial morbidity and mortality, with potentially important public health implications, due to its zoonotic potential. Since the majority of human and animal infections are attributed to contact with urine or through indirect environmental transmission, controlling urinary shedding of leptospires in dogs could be an important step in reducing both human and animal infections [1, 2]. Urinary shedding of leptospira in asymptomatic dog populations has ranged from 1.5–7% [3]. Reduced urinary shedding of leptospira by dogs is primarily achieved is through vaccination [4]. However, as existing leptospirosis vaccines only offer protection towards the specific serovars included in their formulation, or closely related serovars, even with vaccination urinary shedding may not be eliminated [4, 5]. Since over 300 leptospira serovars are recognised, a lack of cross-protection is a key limitation of existing vaccines [6, 7]. Leptospirosis vaccine technology has remained similar since their first commercial use in the 1960s and vaccines that provide cross-protective immunity between serogroups remain elusive [4] The original bivalent (L2), leptospirosis vaccines provided protection against the two serovars, Canicola and Icterohaemorrhagiae, that were historically associated with the canine disease. More recently, tetravalent (L4) vaccines have been licensed in Europe that offer protection against a further two serovars, Grippotyphosa and Bratislava [5, 8]. In the USA, the vaccine offers protection against the Pomona serovar in place of Bratislava [4, 8]. This increased coverage was in response to an increase in cases in mainland Europe being attributed to Grippotyphosa and Bratislava serovars [5, 9–13]. However, the proportion of cases attributed to different serovars may vary between the UK and continental Europe [10–14].

Vaccination against leptospirosis is considered to be part of the ‘core’ vaccination programme for dogs in the UK and is administered annually [15]. However, there is limited evidence on the current level of leptospirosis vaccine uptake by pet owners in the UK. Survey data of veterinary practice by Ball and colleagues (2014) reported 60% of dogs in those clinics surveyed were up to date with their leptospirosis vaccination [16, 17]. Lower vaccine uptake has been associated with various factors such as owner concern for vaccine adverse effects, socio-economic factors and required frequency of administration [18–20]. Although the frequency of suspected vaccine-associated adverse events (VAAEs) reported with leptospirosis vaccines in dogs were found to be comparable to other companion animal vaccines, there remains a level of controversy and scepticism about the leptospirosis vaccine’s safety in some pet-owners and social media groups, which could influence vaccine usage [21, 22].

The aim of the current study was to explore the factors associated with leptospirosis vaccine usage in a first-opinion practice setting to aid understanding of vaccine epidemiology and generate a benchmark for future comparisons. The study objectives were to: [1] describe the incidence of leptospirosis vaccine administration (tetravalent or bivalent) in dogs attending primary care practice in the UK during a 12 month period in 2016 [2]; visualise the spatial variation of leptospirosis vaccine usage across the UK at that time [3]; utilise mixed effects logistic regression models to explore associations between demographic and spatial factors and administration of a leptospirosis vaccine.

## Results

### Study population demographics

The VetCompass population included 905,543 dogs under veterinary care at 886 veterinary clinics during 2016. The most common breeds in the study population were Labrador Retrievers (*n* = 58,768, 6.6%), Staffordshire Bull Terriers (*n* = 51,883,5.8%), Jack Russell Terriers (*n* = 47,537,5.3%), Shih-Tzus (*n* = 32,340,3.6%) and Cocker Spaniels (*n* = 31,554,3.5%). Crossbreeds were the most common overall (*n* = 190,242, 21.3%). The study population comprised of more dogs from KC recognised breeds (*n* = 626,299,70.4%) than non-KC recognised dogs (*n* = 258,834, 29.1%). The most frequently recorded KC recognised breed groups were: Terrier (*n* = 143,038, 16.1%), Toy (*n* = 129,719, 14.6%) and Gundogs (*n* = 133,082, 15.0%). There were more males (*n* = 461,215, 50.9%) than females (*n* = 423,760, 46.7%) in the study population. Dogs were more frequently recorded as entire (*n* = 484,166, 53.5%) than neutered (*n* = 400,811, 44.3%). The median age of dogs in the denominator population was 4.21 years (IQR = 1.567–7.87). Owner postcodes of dogs in the study population were most frequently classified as the most deprived quintile of IMD rankings (*n* = 185,408, 20.9%) and least frequently in the least deprived quintile of IMD rankings (*n* = 111,448, 12.3%). Veterinary clinics attended by the study population were most frequently classified as the second most deprived quintile of IMD rankings (*n* = 299,944, 33.7%).

### Estimated uptake of leptospirosis vaccination in dogs under veterinary care at VetCompass primary care practices

Within the study population, 49.0% (*n* = 443,716 / 905,543, 95%CI 48.9–49.1%) dogs had a record of receiving at least one leptospirosis vaccine during 2016. Of the dogs with a recorded leptospirosis vaccination, 77.8% of EPRs (345,164/443,716, 95%CI 77.6–77.8%) specifically reported the level of serovar coverage provided by the vaccine administered. The estimated proportional uptake of a bivalent vaccine was 11.0% (*n* = 99,366/905,543, 95% CI 10.5–12%). whilst the uptake of a tetravalent vaccine was 27.1% (*n* = 245,798/905,543, 95%CI 26.5–27.6%).

### Spatial distribution of leptospirosis vaccine usage

**Fig. 1:**
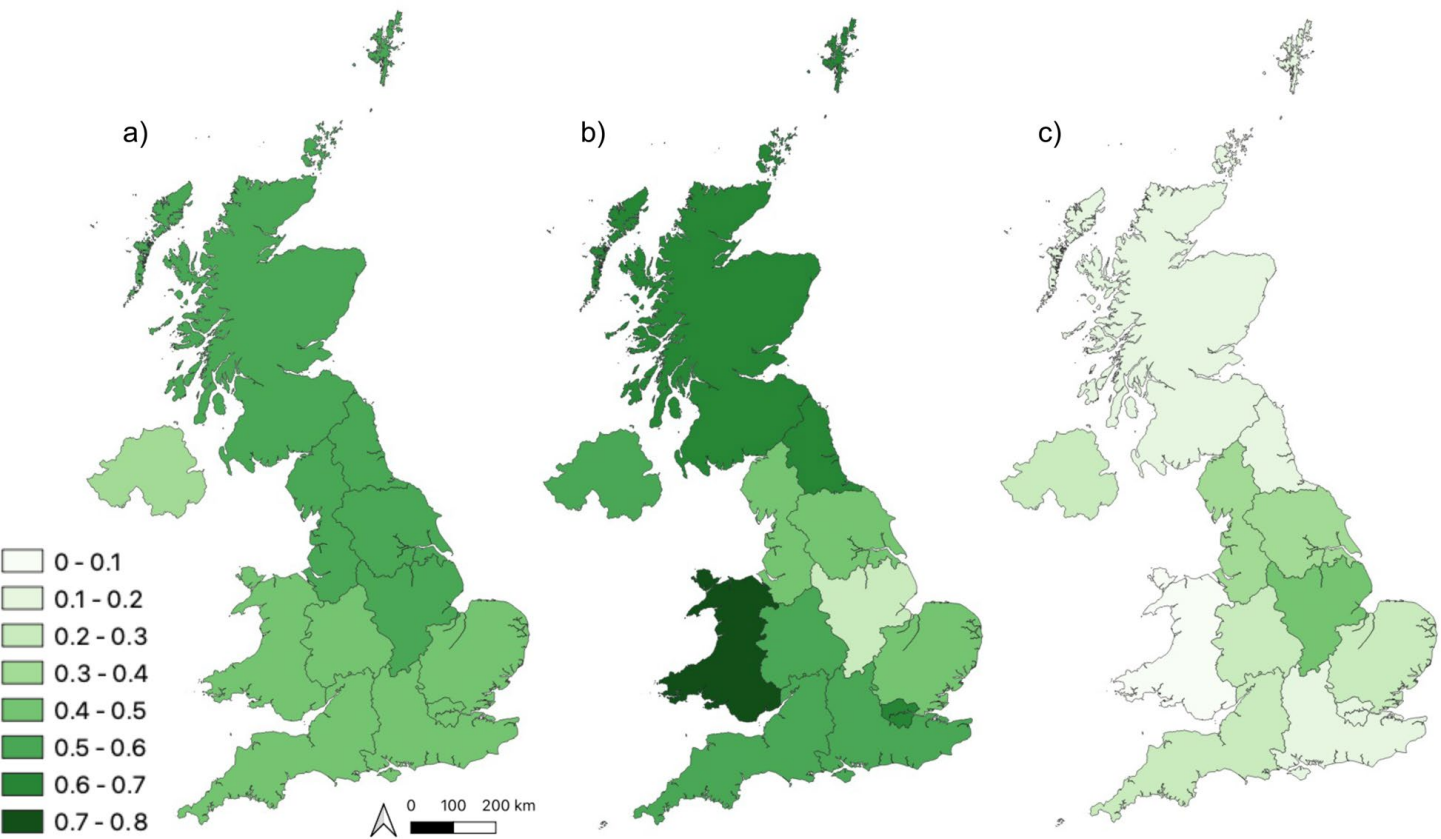
Choropleth maps indicating the proportion of dogs vaccinated for leptospirosis in 2016 in each region. Maps are at a NUTS level 1 region resolution. Map **a**) indicates the proportion of dogs in each region that are vaccinated against leptospirosis (with a leptospirosis vaccine of any level of serovar coverage), **b**) indicates the proportion of dogs in the regions that received a tetravalent leptospirosis vaccine and **c**) indicates the proportion of dogs in the regions that received a bivalent leptospirosis vaccine. Areas in darker green indicate higher proportion of vaccinated dogs

Across the 12 regions of the UK, the proportion of dogs vaccinated against leptospirosis varied substantially between the region with the highest proportion of leptospirosis vaccinations versus the lowest (Chi square = 573, p = < 0.001) (Fig. 1a). Leptospirosis vaccine administration was highest in the North East of England (56.5%) and lowest in Northern Ireland (38.1%). Proportional usage of bivalent or tetravalent leptospirosis vaccine varied significantly between regions of highest and lowest usage for both types of vaccine (Bivalent chi square = 2867.5, *p* < 0.001 and tetravalent chi square = 2754.5, p < 0.001; Fig. 1b and c). The relatively greater usage of tetravalent vaccines versus a bivalent product was most noticeable in Wales (71.2% L4 vs 7.1% L2), but the opposite was the case in the East Midlands (26.7% L4 vs 43.7% L2).

### Factors associated with leptospirosis vaccine administration

**Fig. 2:**
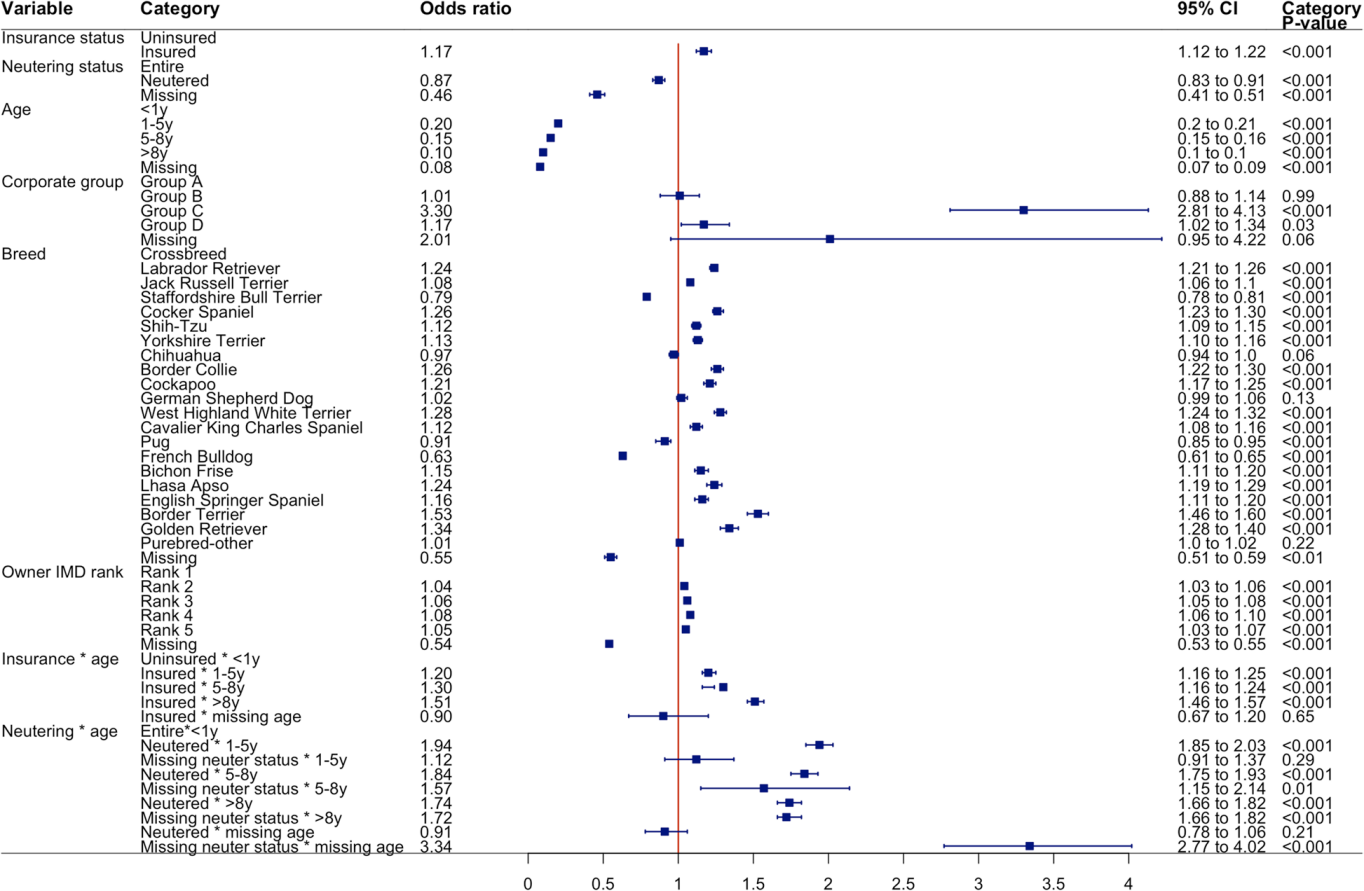
Results of mixed effects model examining significant variables associated with leptospirosis vaccination administration for dogs under primary veterinary care in 2016. The final model included neutering status, insurance status, top 20 breeds, owner IMD rank, biological age and corporate group. Interaction terms between age and neutering and age and insurance are shown. Missing data was retained and is labelled ‘missing’ here

All tested variables were liberally associated (*p* < 0.2) with leptospirosis vaccine status in univariable mixed effects logistic regression with Clinic ID as a random effect (Table 1). The final breed-focused model retained 6 independent variables (Fig. 2). Due to collinearity, a separate model with Kennel Club group and weight was evaluated in place of breed (Supplementary Material, Fig. S1). All adult dogs had reduced odds of receiving a leptospirosis vaccination compared to the baseline of dogs < 1 year old. Dogs > 8 years old had lowest odds (OR = 0.08, 95%CI 0.07–0.09) of any group. Dogs had overall increased odds of receiving a leptospirosis vaccination if they were insured (OR = 1.22, 95%CI = 1.17–1.28). However, there was an interaction between age and insurance, such that adult insured dogs of all age brackets had increased odds of receiving a leptospirosis vaccination versus a baseline of uninsured dogs less than 1 year old. This was highest in insured dogs over 8 years old (OR = 1.58, 95% CI = 1.50–1.67) of vaccination. Neutered dogs had lower odds of receiving a vaccination than entire dogs (OR = 0.87, 95%CI 0.83–0.91). However, there was an interaction between age and neutering status such that neutered dogs of all age groups >1y had increased odds of vaccination when compared to the baseline of entire dogs less than 1 year old. Odds were highest for neutered dogs between 1 and 5 years old (OR = 1.94, 95%CI = 1.85–2.03). After accounting for other explanatory variables, fourteen of the breed types examined had increased odds of vaccination compared to the baseline of crossbred dogs. The breed types at highest odds of receiving a leptospirosis vaccination were: Border Terriers (OR = 1.49, 95%CI 1.42–1.57), Golden Retrievers (OR = 1.30, 95%CI = 1.24–1.37), Cocker Spaniels (OR = 1.27, 95%CI 1.23–1.31) and West Highland White Terriers (OR = 1.27, 95%CI 1.22–1.31). French Bulldogs (OR = 0.64, 95%CI = 0.62–0.67), Staffordshire Bull Terriers (OR = 0.79, 95%CI 0.78–0.82) and Pugs (OR = 0.91, 95%CI =0.88–0.95) had significantly reduced odds of receiving a leptospirosis vaccination when compared to crossbred dogs. Dogs from less deprived areas (those within IMD quintiles 2–5) all had slightly increased odds of receiving a leptospirosis vaccination compared to dogs from the most deprived areas of Great Britain (IMD = 1). Veterinary group was retained to adjust for the inherent structure of the data and odds of receiving a leptospirosis vaccine was variable between corporate groups. Dogs attending two of the four veterinary groups had significantly increased odds of receiving a leptospirosis vaccination versus the baseline group of dogs attending Group A.

**Table 1:** Descriptive statistics of the study population and variables examined in univariable mixed effect model analysis

Variable	Category	Leptospirosis vaccinated	Leptospirosis unvaccinated	OR (95% CI)	***p*** value
**Sex**	Female	204,335 (47.0)	219,425 (48.3)	Baseline	< 0.001
	Male	229,215 (52.7)	232,000 (51.1)	1.06 (1.05–1.07)	< 0.010
	Not recorded	1556 (0.4)	2509 (0.6)	0.71 (0.66–0.76)	< 0.001
**Neutering**	Entire	232,152 (53.4)	252,014 (55.5)	Baseline	< 0.001
	Neutered	201,399 (46.3)	199,412 (43.9)	1.10(1.09–1.11)	< 0.001
	Not recorded	1555 (0.4)	2508 (0.6)	0.71 (0.66–0.75)	< 0.001
**Sex-neuter**	Female Entire	106,953 (24.6)	122,389 (27.0)	Baseline	< 0.001
	Female Neutered	97,382 (22.4)	97,036 (21.4)	1.15(1.14–1.17)	< 0.001
	Male Entire	125,198 (28.8)	129,624 (28.6)	1.11(1.10–1.12)	< 0.001
	Male Neutered	104,017 (23.9)	102,376 (22.6)	1.17(1.16–1.19)	< 0.001
	Not recorded	1556 (0.4)	2509 (0.6)	0.75 (0.71–0.80)	< 0.001
**Insurance**	Uninsured	370,530 (85.2)	402,314 (88.6)	1.70(1.67–1.73)	< 0.001
	Insured	64,578 (14.8)	51,742 (11.4)	Baseline	< 0.001
**KC or not**	Not KC recognised	128,963 (29.6)	129,871 (28.6)	Baseline	< 0.001
	KC recognised	305,133 (70.1)	321,166 (70.7)	0.95 (0.95–0.96)	< 0.001
	Not recorded	1011 (0.2)	2944 (0.6)	0.36 (0.34–0.39)	< 0.001
**Purebred or not**	Crossbred	91,490 (21.0)	98,752 (21.8)	Baseline	< 0.001
	Designer crossbred	29,588 (6.8)	21,589 (4.8)	1.52(1.49–1.54)	< 0.001
	Purebred	313,018 (71.9)	330,696 (72.8)	1.02(1.02–1.03)	< 0.001
	Not recorded	1011 (0.2)	2944 (0.6)	0.36 (0.34–0.39)	< 0.001
**Common breeds**	Crossbred	91,490 (21.0)	98,752 (21.7)	Baseline	< 0.001
	Labrador Retriever	30,706 (7.1)	28,062 (6.2)	1.17(1.15–1.19)	< 0.001
	Jack Russell Terrier	21,945 (5.0)	25,592 (5.6)	0.92(0.90–0.94)	< 0.001
	Staffordshire bull terrier	20,923 (4.8)	30,960 (6.8)	0.72(0.71–0.73)	< 0.001
	Cocker Spaniel	17,263 (4.0)	14,291 (3.1)	1.28(1.25–1.31)	< 0.001
	Shih-Tzu	16,811 (3.9)	15,529 (3.4)	1.16(1.13–1.19)	< 0.001
	Yorkshire Terrier	13,032 (3.0)	14,610 (3.2)	0.97(0.95–1.00)	< 0.001
	Chihuahua	12,640 (2.9)	12,032 (2.6)	1.13(1.10–1.16)	< 0.001
	Border Collie	11,471 (2.6)	10,460 (2.3)	1.17(1.14–1.20)	< 0.001
	Cockapoo	10,905 (2.5)	7026 (1.5)	1.72(1.67–1.78)	< 0.001
	German Shepherd Dog	10,119 (2.3)	10,860 (2.4)	1.00(0.97–1.05)	0.980
	West Highland White Terrier	9100 (2.1)	9458 (2.1)	1.02(0.99–1.05)	0.190
	Cavalier King Charles Spaniel	8327 (1.9)	8630 (1.9)	1.04(1.01–1.07)	0.012
	Pug	7996 (1.8)	7992 (1.8)	1.11(1.08–1.15)	< 0.001
	French Bulldog	7165 (1.6)	8975 (2.0)	0.92(0.89–0.94)	< 0.001
	Bichon Frise	6633 (1.5)	6454 (1.4)	1.13(1.10–1.18)	< 0.001
	Lhasa Apso	6664 (1.5)	5690 (1.3)	1.17(1.13–1.21)	< 0.001
	Springer Spaniel	5467 (1.3)	5278 (1.2)	1.05(1,01–1.09)	0.016
	Border Terrier	5330 (1.2)	4165 (0.9)	1.25(1.30–1.41)	< 0.001
	Golden Retriever	5060 (1.2)	4580 (1.0)	1.23(1.19–1.30)	< 0.001
	Purebred-other	115,049 (26.4)	121,641 (26.8)	1.04(1.03–1.05)	< 0.001
	Breed not recorded	1012 (0.2)	3019 (0.7)	0.38 (0.36–0.41)	< 0.001
**Kennel Club group**	Not KC Recognised	128,963 (29.6)	129,871 (28.6)	Baseline	< 0.001
	Gundog	69,812 (16.0)	63,270 (13.9)	1.10(1.08–1.11)	< 0.001
	Hound	14,913 (3.4)	15,985 (3.5)	0.96(0.93–0.98)	< 0.001
	Pastoral	25,188 (5.8)	25,510 (5.6)	0.99(0.97–1.01)	0.190
	Terrier	64,794 (14.9)	78,244 (17.2)	0.82(0.81–0.83)	< 0.001
	Toy	63,465 (14.6)	66,254 (14.6)	0.98(0.97–0.99)	0.004
	Utility	49,693 (11.4)	51,229 (11.3)	0.97(0.96–0.99)	< 0.001
	Working	17,268 (4.0)	20,674 (4.6)	0.83(0.82–0.86)	< 0.001
	Not recorded	1011 (0.2)	2944 (0.6)	0.36 (0.34–0.39)	< 0.001
**NUTS region**	North East England	16,559 (3.8)	13,351 (2.9)	Baseline	0.340
	North West England	36,383 (8.4)	40,720 (9.0)	0.89(0.77–1.03)	0.330
	Yorkshire and Humber	35,433 (8.1)	28,499 (6.3)	1.10(0.93–1.30)	0.450
	East Midlands	34,970 (8.0)	31,860 (7.0)	1.06(0.90–1.24)	0.650
	West Midlands	100,497 (23.1)	93,157 (20.5)	0.96(0.82–1.14)	0.770
	East of England	50,888 (11.7)	56,472 (12.4)	1.04(0.91–1.19)	0.720
	London	42,126 (9.7)	49,909 (11.0)	1.15(0.92–1.43)	0.220
	South East England	55,301 (12.7)	66,518 (14.6)	0.92(0.81–1.06)	0.480
	South West England	25,609 (5.9)	32,023 (7.1)	0.84(0.73–0.97)	0.150
	Wales	16,273 (3.7)	19,885 (4.4)	0.84(0.67–1.07)	0.260
	Scotland	18,835 (4.3)	18,009 (4.0)	1.08(0.87–1.34)	0.580
	Northern Ireland	2234 (0.5)	3653 (0.8)	0.79(0.59–1.06)	0.190
	Not recorded	0	0		
**Clinic IMD ranked**	1	88,189 (20.3)	90,225 (20.0)	Baseline	0.150
	2	150,154 (34.5)	149,790 (33.2)	1.01 (0.89–1.15)	0.830
	3	71,017 (16.3)	82,013 (18.2)	1.00 (0.88–1.15)	0.940
	4	63,948 (14.7)	70,477 (15.6)	1.02 (0.89–1.17)	0.780
	5	56,286 (12.9)	55,628 (12.3)	1.15 (1.01–1.32)	0.040
	Not recorded	5514 (1.3)	3120 (0.7)	4.27 (2.11–8.65)	< 0.001
**Owner IMD ranked**	1	93,551 (21.5)	91,857 (20.3)	Baseline	< 0.001
	2	78,351 (18.0)	81,759 (18.1)	1.04 (1.02–1.06)	< 0.001
	3	85,233 (19.6)	85,296 (18.8)	1.07 (1.05–1.09)	< 0.001
	4	83,725 (19.3)	81,688 (18.0)	1.08 (1.06–1.09)	< 0.001
	5	55,973 (12.9)	55,475 (12.3)	1.05 (1.03–1.07)	< 0.001
	Not recorded	37,949 (8.7)	56,543 (12.5)	0.60 (0.58–0.61)	< 0.001
**Vet Group**	A	218,957 (50.3)	252,138 (55.9)	Baseline	< 0.001
	B	23,668 (5.4)	19,028 (4.2)	1.99 (1.71–2.30)	< 0.001
	C	64,942 (14.9)	86,392 (19.1)	1.03 (0.94–1.14)	0.540
	D	124,487 (28.6)	92,946 (20.6)	1.23 (1.11–1.37)	< 0.001
	E	3054 (0.7)	749 (0.2)	2.04(1.15–3.61)	0.020
**Bodyweight (kg) – 10 kg bins**	0- < 10	131,722 (30.3)	133,008 (29.3)	Baseline	< 0.001
	10- < 20	182,679 (42.0)	187,204 (41.2)	0.99(0.98–1.00)	0.110
	20- < 30	65,728 (15.1)	72,403 (16.0)	0.92(0.90–0.93)	< 0.001
	30- < 40	44,429 (10.2).	45,274 (10.0)	0.99(0.97–1.00)	0.100
	> 40	7966 (1.8)	10,766 (2.4)	0.74(0.72–0.77)	< 0.001
	Not recorded	2581 (0.6)	5201 (1.1)	0.71 (0.67–0.75)	< 0.001
**Age**	<1y	95,736 (22.0)	35,070 (7.7)	Baseline	< 0.001
	1-5y	177,115 (40.7)	182,980 (40.4)	0.31(0.30–0.31)	< 0.001
	5-8y	83,467 (19.2)	88,698 (19.6)	0.28(0.28–0.29)	< 0.001
	>8y	76,573 (17.6)	138,056 (30.4)	0.17(0.16–0.17)	< 0.001
	Missing	2192 (0.5)	8666 (1.9)	0.08 (0.08–0.09)	< 0.001

The study population comprised of leptospirosis vaccinated dogs (48.9%,*n* = 435,106) and dogs that did not receive a leptospirosis vaccination (51.7%, *n* = 453,934) from dogs under primary veterinary care in 2016. Missing data were retained and coded as ‘not recorded’. Percentages shown in columns are column percentages

In the second model (Fig. 2, Kennel Club group and weight replacing breed), Gundog (OR = 1.29, 95%CI 1.27 to 1.31) and Pastoral (OR = 1.19, 95%CI 1.16 to 1.21) breed groups had increased odds of receiving a vaccination. As dog bodyweight increased odds of receiving a leptospirosis vaccine decreased. Dogs that weighed over 40 kg had nearly half the odds (OR = 0.62, 95%CI 0.60 to 0.65) of receiving a leptospirosis vaccine when compared to dogs weighing between 0 and 10 kg (Supplementary material, Fig. S1).

For the final multivariable mixed effect models, the ICC was 0.09. This indicated that there was limited clustering of vaccine use at the individual clinic level, even when fixed effects were incorporated.

## Discussion

Despite vaccination being a routine part of veterinary clinical practise, there has been minimal exploration of the proportional usage of leptospirosis vaccines and factors associated with owner compliance in the UK. This study identified that nearly half (49%) of the population of UK dogs under primary care were vaccinated during 2016, with a tetravalent (L4) leptospirosis vaccine administered more frequently (27.1%) than a bivalent (L2) vaccine (11%). Adult dogs had between a five (1-8y old) and tenfold (>8y old) reduction in odds for receiving a leptospirosis vaccination compared to dogs less than 1 y old. Neutered and uninsured dogs had reduced odds of leptospirosis vaccination. Odds of vaccine administration were increased in certain breeds (for example, Border Terrier, Golden Retriever) and reduced in others (French Bulldog, Staffordshire Bull Terrier).

There are relatively few published studies that estimate vaccine uptake in UK dogs. Of the two studies that have explored leptospirosis vaccine administration in the UK, both reported higher rates of uptake than was seen here (60.4 and 95.5%) [16, 18]. However, the methodologies used in those two reports were substantially different from the study undertaken here, with one being a survey with a relatively low response rate (18.9%) and the other reporting the proportion of dogs who had ever received a leptospirosis vaccine administration over their lifetime [16, 18]. A survey of dogs in Germany found comparable leptospirosis vaccine coverage to the present study (50.1 vs 49%) [20]. Since licenced canine leptospirosis vaccines are killed vaccines, they are unlikely to have a duration of immunity (DOI) that is substantially longer than the reported 12 months, although one study identified immunity lasting at least 15 months to the Grippotyphosa serovar [23].

Age was the variable that was the greatest predictor of leptospirosis vaccination in 2016. Dogs from the oldest age group (> 8 years old) were over 10 times less likely to have received a vaccine against leptospirosis than dogs < 1 year old. Vaccine uptake is typically higher for primary courses than booster doses [19, 20, 24–27]. This may reflect novelty of a new pet, scheduling effort for an annual vaccine, cost concerns or perceived immunity from the initial vaccine course [19, 20]. Although modified live virus vaccines may confer a longer, or even lifelong, immunity than the manufacturer’s DOI,this is likely not the case for killed vaccines such as the leptospirosis vaccine [28]. The move away from routine annual vaccination to a three yearly schedule for MLV vaccines may mean that owners do not consider an annual vaccine (e.g. for leptospirosis) to be necessary. Clarifying this potential misconception with owners may be important. Differences between primary and booster course uptake were not explored in the current analyses, due to variability in the veterinary group recording systems.

Previous studies have reported reduced odds of a leptospirosis diagnosis in all adult dogs versus dogs < 1 year, with lowest odds in geriatric dogs (> 8) [29]*.* Reduced odds of leptospirosis diagnosis and (counterintuitively) vaccine administration in older dogs might suggest that leptospirosis is actually an infectious disease that is of lower concern in older dogs. This could reflect older animals experiencing reduced environmental exposure, with fewer walks or walks in different environments, or gaining a greater degree of immunity through natural exposure acquired throughout their lifetime. Alternatively, it may indicate that owners are less likely to pursue diagnostics or booster vaccines in older dogs [20, 29]. In contrast, amongst the subset of insured dogs in this study, older insured dogs showed increased odds of leptospirosis vaccination. Similarly, although neutered dogs had reduced odds of receiving a leptospirosis vaccination when the interaction between age and neutering status was explored, adult neutered dogs had increased odds of vaccination. These interactions indicate that vaccine uptake in older dogs is somewhat variable, and that neutering and insurance status may reflect a higher level of owner engagement with preventative healthcare and hence increased vaccination.

Breed associations with leptospirosis vaccination varied. Many of the breeds examined here had increased odds of leptospirosis vaccination compared with cross-breeds, with highest odds seen in Border Terriers, Golden Retrievers, Cocker Spaniels, Border Collies and Labrador Retrievers. In contrast, French Bulldogs, Staffordshire Bull Terriers and Pugs were associated with reduced odds of receiving a leptospirosis vaccination. Several of the breeds associated with increased odds of receiving a leptospirosis vaccine were the same as those identified in a previous study with increased odds of a diagnosis of leptospirosis (i.e., English Springer Spaniel, Border Collie, Labrador Retriever) [29]. Leptospirosis has historically been regarded as a disease of working or hunting dogs reportedly due to increased exposure to contaminated environmental sources [14, 29–31]. Consequentially, this perception may have led to increased owner/veterinarian awareness of this disease in these particular breed groups and consequently an increased recommendation and uptake of prevention strategies such as vaccination and diagnostic testing in these breed groups. Additionally, the reduced odds of leptospirosis vaccine administration in the Toy breed group (in the KC breed group model, S1) and certain brachycephalic breeds might reflect concerns over increased risk of adverse vaccine reactions reported in Toy breeds and some brachycephalic breeds [32–34].

Breed type may act as a proxy for owner socioeconomic status. For example, rural areas have typically lower levels of deprivation and higher levels of dog ownership [35, 36]. Larger breeds may be more popular in rural areas due to increased space demands. Human vaccination uptake studies typically find strong associations between socioeconomic status and vaccine usage, with generally positive associations between higher vaccine uptake and higher socioeconomic status reported [37–39]. In this study we found only a small increase in odds of leptospirosis vaccination as owner socioeconomic status increased. Clinic IMD rank was not retained in the final models, potentially indicating that factors other than socioeconomic status drive clinic choice. Due to the mechanism of the VetCompass data capture process, IMD was only able to be explored at owner partial or clinic postcode level whereas typically human studies look at socioeconomic variables at the individual or household resolution [38, 39]. A previous EPR study that used complete owner postcode level IMD data identified a larger, positive association between reduced deprivation and increased vaccine uptake than seen here [18]. Coarser aggregation of socioeconomic data may weaken the association between socioeconomic status and dog vaccination uptake.

Individual clinic IDs were used as the random effect in this mixed effect model analyses. However, individual veterinarian behaviour may also have been an important random effect to include. It is unclear how much impact individual veterinarians might have on vaccine administration or whether policy is set at a clinic or corporate level. Some work suggests the influence of the veterinarian has a large impact on owner’s vaccine decision making [20, 27]. Unfortunately, due to database limitations exploration of this veterinarian effect was not possible.

The study population here was relatively large (~ 900,000 dogs) and represents a sample from approximately 30% of UK first opinion veterinary practices. As VetCompass largely receives its data from the major veterinary corporate groups there are some areas of the country, with fewer corporate owned clinics and fewer clinics overall, that are underrepresented. These are largely rural areas in Wales, Scotland and Northern Ireland. Therefore, risk or protective factors for vaccination identified in this research may not necessarily be applicable to dogs from these underrepresented areas. Additionally, vaccine usage was estimated here using a study population containing only dogs under veterinary-care either in 2016 itself and/or in both2015 and 2017. It is possible that this study population may differ from the overall UK dog population, which has been estimated to be between 8 and 12 million [26, 40].

Unfortunately, due to database limitations exploration of veterinarian effect was not possible. The models built for these analyses indicated that vaccine usage had limited clustering at the clinic level, suggesting that the demographic variables explored here do not adequately capture all factors influencing vaccination decision making. Qualitative studies in Germany have explored the impact of the client’s relationship with their veterinarian and how this affects their choice of information source on vaccination [20, 27]. Similar exploration could be undertaken on dog owning clients in the UK to assess the importance of these variables.

Inclusion or exclusion of missing data was also explored (models with exclusion of missing data not shown here) with the inclusion of missing data model having a slight improvement in fit to data (Pseudo R2 0.21 versus 0.19). Missing data were a significant category for all of the independent variables. Due to their significance as a category and its inclusion leading to a slight improvement in model fit, the models built with missing data retained were deemed to be better final model choices. Both the missing excluded and missing retained models kept the same independent variables and minimal differences in magnitude of association with factors were seen.

This study explored leptospirosis vaccine usage during a single period of time (2016), so it remains unclear how leptospirosis vaccine administration and factors associated with administration will have changed over time, particularly during the Covid-19 pandemic. It is also unclear how the cost-of-living increase at the time of writing (2022) will affect decision making of pet owners in terms of veterinary / preventative healthcare. Reports commissioned by the Peoples Dispensary for Sick Animals (PDSA) indicate there is a general trend of reduced uptake of both primary and booster courses of vaccines [26], which might be identified in the VetCompass database through exploration of more recent time points. Conversely, it is possible that external factors such as confirmation of the safety of the tetravalent vaccine may have a positive impact on vaccine uptake [21]. Additionally, comparison with other dog vaccines (e.g. canine parvovirus) would allow investigation of whether the factors associated in these analyses are unique to leptospirosis vaccination or are applicable more widely to dog vaccinations overall. Future studies could also assess for associations between regional vaccine usage and disease burden.

## Conclusion

This study provides a robust estimate of proportional leptospirosis vaccine usage (49%) in dogs under primary care in the UK in 2016 and identifies several demographic variables associated with leptospirosis vaccine administration during this period. Further work determining leptospirosis vaccine incidence at other time points and exploration of other factors associated with owner decision making in terms of vaccine administration are warranted.

## Materials and methods

### Study population

Ethical approval for the study was granted by the RVC Social Science Research Ethical Review Board (SR2018–1652). The VetCompass Programme collates de-identified electronic patient record (EPR) data from participating primary-care veterinary practices in the UK for epidemiological research [41]. All methods were carried out in accordance with relevant ethical guidelines and regulations.

A cohort study design was used to estimate incidence of leptospirosis vaccination in the 2016 cohort and assess factors associated with vaccine uptake in dogs under veterinary care within the dataset. Dogs under veterinary care were defined as those with either a) ≥1 EPR (VeNom diagnosis term, free-text clinical note, treatment or bodyweight) recorded during 2016 and/or b) ≥1 EPR recorded during both years on either side of 2016. Patient records consisted of a unique ID number alongside species, breed, sex, neutering status, insurance status, age and bodyweight. Additionally, patient records contained veterinary clinic full postcodes and owner home partial postcodes. Clinical information and treatments administered were recorded as free-text and/or billable items. A list of leptospirosis vaccine-related terms was generated to search the database, using the British Small Animal Veterinary Association (BSAVA) Formulary, the National Office for Animal Health (NOAH) compendium and a list of any treatments recorded in the VetCompass 2016 denominator (Supplementary material, Table S1). All patients with at least one treatment administered that corresponded to any of these terms were identified. Where possible vaccine terms were further categorised according to the number of leptospira serovars they contained (i.e. bivalent or tetravalent). Since the stated duration of immunity (DOI) of all licensed leptospirosis vaccines is reported to be 12 months, a dog was only classified as vaccinated if any item from the vaccine treatment list had been administered between 1st January and 31st December 2016 [8]. Additionally, for dogs less than 1 year old, two leptospirosis vaccinations needed to be recorded in this time period to represent a full primary course. All other dogs were classified as not vaccinated for the purpose of the risk factor analysis. Sample size calculations estimates indicated that between 389 and 7964 dogs vaccinated against leptospirosis and 389–7964 dogs not vaccinated against leptospirosis would be required to detect a risk factor with an odds ratio of 1.5, with exposures in the non-vaccinated group of 1 to 40% (based on estimates that 40% of dogs are neutered, 10% of dogs would be > 8 years old and each of the most common breeds would represent 1% of dogs, confidence level 95%, power 80% [42].

Incidence risk of leptospirosis vaccination was estimated from the number of dogs with a leptospirosis vaccination item recorded in their treatment notes in 2016 divided by the overall number of dogs under veterinary care in 2016. The 95 % confidence interval (95% CI) was estimated using exact methods [43].

Risk factor information for dogs that were leptospirosis vaccinated and not leptospirosis vaccinated during 2016 included: breed, Kennel Club group, age, neutering status, sex, weight, insurance status, owner socioeconomic demographics, region and socioeconomic demographics of clinic, veterinary clinic and veterinary corporate group.

Breed information recorded in VetCompass were cleaned and mapped to a VetCompass and VeNom breed list (VeNom Veterinary Nomeclature) [44]. A purebred variable grouped recognised breeds as ‘purebred’, dogs with a ‘breed’ name contrived from two or more purebred terms as ‘designer crossbred’ and all others as ‘crossbred’ [45]. Purebreds were further categorized by UK Kennel Club (KC) breed-recognition (recognised/not recognised) and KC breed group (Gundog, Hound, Pastoral, Terrier, Toy, Utility, Working) [45]. To ensure sufficient power for analysis, only the 20 most frequently recorded breed types in the 2016 denominator population were examined individually. The most common breed types included both purebred dogs and designer crossbreed types. Any dogs with a recorded breed that was not one of the 20 most frequent was recorded as ‘purebred-other’. Dogs without breed information available were excluded from breed related analysis but retained for univariable analysis of other risk factors. Age (years) was defined as the age at first administration of a leptospirosis vaccination in 2016. For the VetCompass dogs that did not receive a leptospirosis vaccination, age was the age (years) on December 31 2016, which was the final date by which these dogs had not been vaccinated against leptospirosis. Age was summarised with median and interquartile range (IQR) and then examined additionally as a categorical variable. Age was examined via biologically relevant division categories: 0-1y, > 1-5y, > 5-8y and > 8 years. Neutering status (‘Entire’, ‘Neutered’ or ‘Not recorded) was the status in the final VetCompass EPR on December 31st 2016 for non-leptospirosis vaccinated dogs and the recorded neutering status when the leptospirosis vaccination was administered. Sex was encoded as ‘Female’, ‘Male’ or ‘Not recorded’ and sex-neutering status was encoded as ‘Female Entire’, ‘Female Neutered’, ‘Male Entire’ or ‘Male Neutered’ or ‘Not recorded’. Bodyweight was recorded in kilograms and was defined as the maximum bodyweight recorded for each dog after being aged > 18 months. Bodyweight was examined in ten kilogram increments. Where available, insurance status of dogs was identified. Insured dogs were those with prior or current evidence of insurance in the EPR and otherwise dogs were classified as uninsured.

Veterinary practice postcodes associated with VetCompass dogs were linked to their corresponding Lower Super Output Areas (LSOA). Owner home partial postcodes associated with VetCompass dogs were at either postcode district or sector resolution and were assigned either LSOA and Local Authority District (LAD) areas, depending on the length of partial postcode provided. Both owner and practice postcodes were assigned their Nomenclature of Units for Territorial Statistics (NUTS) level 1 and 3 region codes. Each LSOA in the UK has an Indices of Multiple Deprivation (IMD) rank value [35, 46]. However, due to IMD ranking being performed using different criteria and indices in countries within the UK, the rank values are not directly comparable. Therefore, the IMD ranks for all LSOA/LAD within individual countries (England, Scotland, Wales, Northern Ireland) were split into quintiles and then assigned a category of 1 (most deprived) to 5 (least deprived) to each patient’s IMD. This allowed for comparison across the UK overall rather than only at a country level. This categorisation of IMD ranking was also performed in a vaccine usage study by Sánchez-Vizcaíno et al., (2018). IMD ranking is generated through the weighting of 37 indicators across different aspects of deprivation such as employment, health, education, crime and housing [35].

### Risk factor analysis

Since vaccine usage was suspected to cluster to some degree at the clinic level, a mixed effects logistic regression model approach was utilised. In this study, clinic ID was set as a random effect. Fixed effects were a mixture of demographic and spatial independent variables. Initial univariable analysis using mixed effect models assessed for potentially significant independent variables. Independent variables with liberal associations (*p* < 0.2) were retained for consideration in multivariable mixed effect models regression. Collinearity of variables evaluated in the multivariable model was assessed through evaluation of the correlation matrices, the Variance Inflation Factor (VIF) and tolerance [47] Where two variables were highly related, the variable considered the most biologically important was retained for consideration. Due to KC breed group and breed variables being highly collinear, two separate models were generated in the multivariable analysis. Separate models were generated with the inclusion or exclusion of missing data and performance was compared to determine whether retention of missing data improved or decreased model performance. Only models with missing data included were reported here.

For the final multivariable models, a manual stepwise backwards elimination model-building approach was adopted. Variables were retained if *p* < 0.05 from the Likelihood Ratio Test (LRT). All potential biologically relevant pairwise interaction terms between the most important independent variable and other independent variables were explored and included in the final model depending on the LRT statistical significance and lower Akaike Information Criterion (AIC) values. Confounding was assessed through addition of each independent variable in a stepwise manner to the model and assessing for substantial (> 20%) increase in odds ratio (OR) when each new variable was added to the model ..(Dohoo et al., 2009). Final model performance was assessed by AUC (Area Under the Curve) of the ROC (Receiver Operator Characteristics). Results were reported as an odds ratio with 95% confidence intervals (CI). Statistical significance was set at *p* < 0.05.

An intraclass correlation coefficient (ICC) was calculated for each mixed effect model to determine compare the variance within a clinic to variance between clinics [48]. Values range from 0 to 1, with higher values indicating a greater magnitude of clustering between clinics [48].

Data cleaning and mixed effects model analysis were performed in RStudio 3.5.1 using the tidyr and lme4 packages [49]. Graphs were generated using the ggplot2 package, AUC and ROC curves were generated in the pROC package and pseudo R2 values were determined using the MuMIN package [49]. Point maps and all choropleths of clinic locations and regional vaccine usage, spatial joins for clinic and owner postcodes to LSOA, LAD codes were performed in QGIS v3.40 [50].

## Supplementary Information

**Additional file 1.**

**Additional file 2: Table S1.** Complete list of possible leptospirosis vaccine related treatment terms used to query the VetCompass database and identify leptospirosis vaccinated dogs in the 2016 denominator population.

## Data Availability

A subset of the datasets generated during and/or analysed during the current study will be made available at the RVC Research Online repository.

## Abbreviations

L2: Bivalent leptospirosis vaccine
L4: Tetravalent leptospirosis vaccine
VAAEs: Vaccine associated adverse events
EPR: Electronic patient record
DOI: Duration of immunity
KC: Kennel Club
IQR: Interquartile range
LSOA: Lower Super Output Area
LAD: Local Authority District
NUTS: Nomenclature unit of territorial statistics
IMD: Indices of multiple deprivation
VIF: Variance inflation factor
LRT: Likelihood ratio test
AIC: Aikake’s information criterion
OR: Odds ratio
AUC: Area under curve
ROC: Receiver operator curve
CI: Confidence interval
ICC: Intraclass correlation coefficient
PDSA: People’s dispensary for sick animals
