# Supplementary figures and images for: Leptospirosis vaccination in dogs attending UK primary care practices: vaccine uptake and factors associated with administration
Source: BMC Vet Res. 2022 Jul 22;18:285. doi: 10.1186/s12917-022-03382-6 (PMC9303131; doi:10.1186/s12917-022-03382-6)

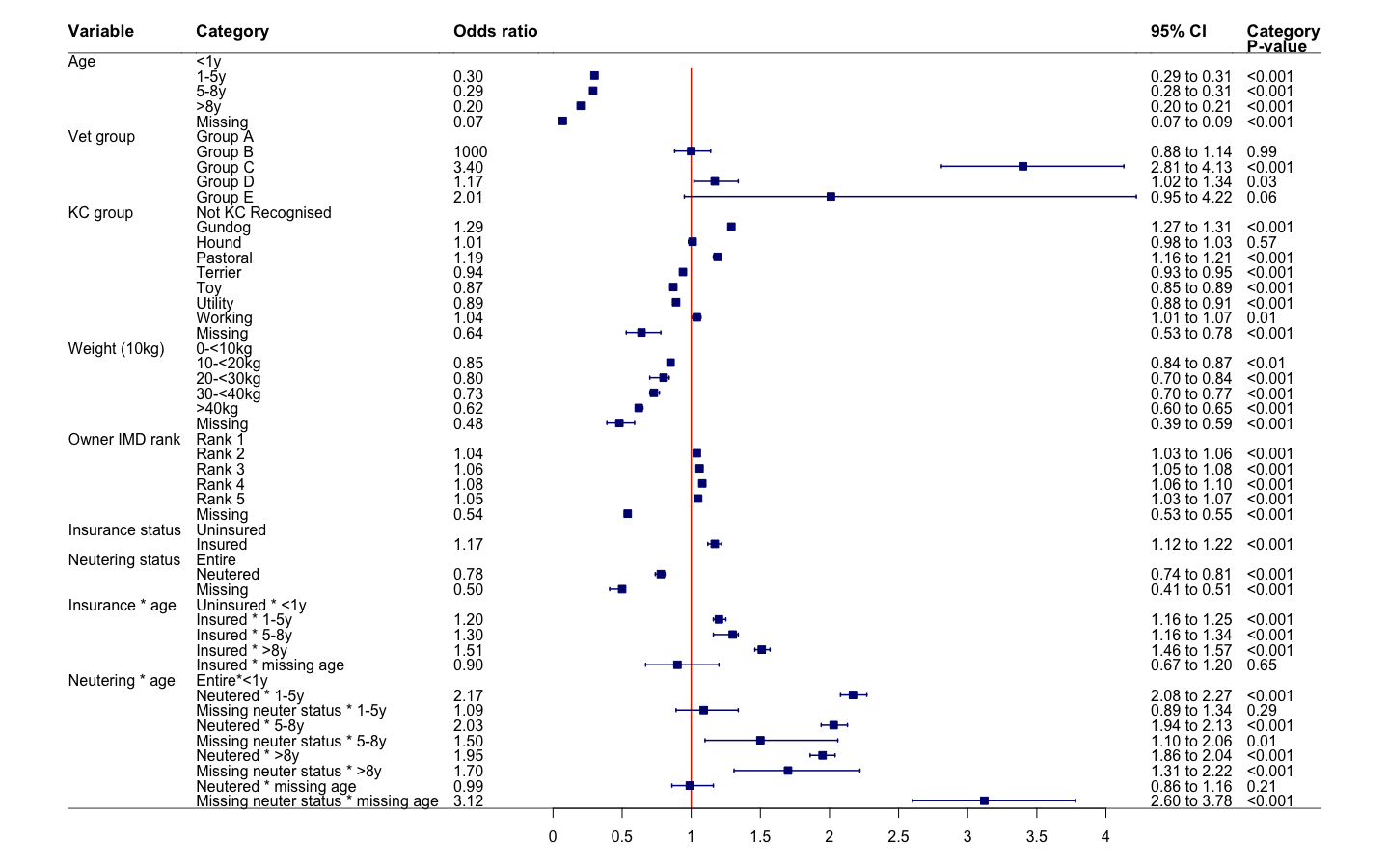

Supplement: Supplementary file 1 — Additional file 1. [file 12917_2022_3382_MOESM1_ESM.png]
