# Supplementary material for: Leptospirosis vaccination in dogs attending UK primary care practices: vaccine uptake and factors associated with administration
Source: BMC Vet Res. 2022 Jul 22;18:285. doi: 10.1186/s12917-022-03382-6 (PMC9303131; doi:10.1186/s12917-022-03382-6)
Supplement: Supplementary file 2 — Additional file 2: Table S1. Complete list of possible leptospirosis vaccine related treatment terms used to query the VetCompass database and identify leptospirosis vaccinated dogs in the 2016 denominator population. [file 12917_2022_3382_MOESM2_ESM.docx]

**Table S1- Complete list of possible leptospirosis vaccine related treatment terms used to query the VetCompass database and identify leptospirosis vaccinated dogs in the 2016 denominator population**

| Vacc Dog Vanguard 7 Amnesty | Vacc 2wk Lepto |
| --- | --- |
| 1st vacc OOD DHP+L4 | Vacc adult 2nd (L4 + DHP) |
| 2nd L4 | Vacc Amnesty DHP (Pi)L 2nd |
| 2nd L4 4 weeks supplement | Vacc Amnesty DHP(Pi)L 1st |
| 2nd Leopto 4 in 4 weeks- prepaid | Vacc Amnesty L2 2nd |
| 2nd lepto | Vacc Amnesty L4 2nd |
| 2nd lepto 4 | vacc booster with 1st and 2nd Lepto and KC |
| 2nd Lepto vaccination | Vacc Charity Dog DHPL |
| 2nd Lepto4 DHP vac | Vacc Charity Dog Lepto4 |
| 2nd letpo | Vacc DHP L4 |
| 2nd Vac L4 | Vacc Dog 1st (DHP/DHPPi/L) |
| 2nd vac. DHP+L4+KC | Vacc Dog 1st Lepto 4 |
| 2nd vacc Nobivac Lepto 4 | Vacc Dog 2nd (DHP/DHPPi/L) |
| 2nd vaccination DHP+L4+KC | Vacc Dog 2nd L4 |
| Booster lepto | Vacc Dog 2nd Lepto |
| Canigen L2 (50) (per Dose) | Vacc Dog 2nd Lepto 4 |
| Canigen Lepto 2 | Vacc Dog 2nd Lepto 4 (Primary) |
| Canigen Lepto 2 (50) | Vacc Dog Booster DHP+L |
| DHPPi +L | Vacc Dog DHP/DHPPi/L Booster |
| Dog 2nd L4 vaccination free of charge | Vacc Dog DHPL4 Booster |
| Dog Annual Health Check & Vacc'N Dhp+l | Vacc Dog L4 2nd Dose |
| Dog Annual Health Check & Vacc'N Dhppi + L | Vacc Dog L4 Booster |
| Dog Annual Health Check & Vaccination L | Vacc Dog Lapsed DHP+ L 1st (course) |
| Dog Annual Health Check & Vaccination Pi+l | Vacc Dog Lapsed L only 2nd FOC |
| Dog Primary1 Health Check & Vacc'N Dhp + L | Vacc Dog Lapsed L4 1st (course) |
| Dog Primary1 Health Check & Vacc'N Dhppi+l | Vacc Dog Lepto booster |
| Dog Primary1 Health Check & Vacc'N L | Vacc Dog Lepto 4 Additional |
| Dog Primary2 Health Check & Vacc'N Dhppi+l | Vacc Dog Nobivac 1st / Course DHP + L2 |
| Dog Primary2 Vaccination Dhp+l | Vacc Dog Nobivac 1st / Course DHP + L4 |
| Dog primary2 vaccination DHP+L | Vacc Dog Nobivac 1st / Course L4 |
| Dog Primary2 Vaccination Dhppi+l | Vacc Dog Nobivac 2nd / Course DHP + L4 |
| Dog Primary2 Vaccination Dhppi+l | Vacc Dog Nobivac 2nd / Course DHP |
| Dog Primary2 Vaccination L | Vacc Dog Nobivac 2nd / Course DHP + L2 |
| Dog Primary2 Vaccination Pi+l | Vacc Dog Nobivac 2nd / Course L4 |
| Dog Primary3 Vaccination L | Vacc Dog Nobivac 3rd L4 |
| L2 | Vacc Dog Nobivac Adult Course DHP + L4 |
| L4 | Vacc Dog Nobivac Amnesty DHP + L4 |
| l4 booster | Vacc Dog Nobivac Booster DHP + L2 |
| lepto 2 | Vacc Dog Nobivac Booster DHP + L4 |
| LEPTO 2 | Vacc Dog Nobivac Booster L2 |
| lepto 4 | Vacc Dog Nobivac Booster L4 |
| lepto booster | Vacc Dog Nobivac DHP + Lepto 4 Booster |
| lepto vacc- amnesty | Vacc Dog Nobivac DHP + Lepto 4 Booster OLD |
| Leptospira | Vacc Dog Nobivac L4 Amnesty |
| Leptospira vaccination | Vacc Dog Vanguard 7 1st (Course) |
| leptospira vaccine | Vacc Dog Vanguard 7 2nd |
| Nobi-Vac Lepto 2 (10) (per Dose) | Vacc Dog Vanguard 7 Adult Course |
| Nobi-Vac Lepto 2 (50) (per Dose) | Vacc Dog Vanguard 7 Amnesty |
| Nobi-Vac Lepto 4 (10) (per Dose) | Vacc Dog Vanguard 7 Booster |
| Nobi-Vac Lepto 4 (50) (per Dose) | Vacc Dog Vanguard 7 Lepto Ci 2nd |
| Nobivac DHPL booster | Vacc Dog Vanguard Lepto Ci Booster |
| nobivac L2 | vacc l4/dhp +KC |
| Nobivac L4 | vacc puppy package 1st & 2nd inj inclusive |
| NOBIVAC L4 (50 DOSE) | Vacc Puppy Primary Vaccination L4 |
| nobivac lepto 2 | Vaccination CK Dog 1st Vanguard 7 |
| Nobivac Lepto 2 (10 box) | Vaccination CK Dog 2nd Vanguard 7 |
| nobivac lepto 2 (10d) | Vaccination CK Dog Booster Vanguard 7 |
| nobivac lepto 2 (50d) | Vaccination CK Dog Nobivac 1st / Course DHP + L4 |
| Nobivac Lepto 2 Vaccine (10) | Vaccination CK Dog Nobivac 2nd / Course DHP + L4 |
| Nobivac Lepto 2 Vaccine (50) | Vaccination CK Dog Nobivac Booster DHP + L4 |
| Nobivac Lepto 4 | Vaccination DT Booster Vanguard 7 |
| Nobivac Lepto 4 | Vaccination DT Booster Vanguard Lepto Ci |
| Nobivac Lepto 4 (10 box) | Vaccination HMP Dog Vanguard 7 |
| nobivac lepto 4 (10d) | Vaccination HMP Dog Vanguard Lepto Ci |
| Nobivac Lepto 4 (50 box) | Vaccination L4+KC |
| nobivac lepto 4 (50d) | Vaccination puppy 1st (DHP + L4 + KC) |
| Nobivac lepto 4 vac | vaccination puppy 1st (DHP + L4) |
| Nobivac Lepto 4 Vaccine (50) | Vaccination Puppy Single (C&C) |
| Nobivac Lepto 4 Vaccine (10) | Vaccination second L4 + DHP (nobivac) |
| Nobivac Lepto4 | vaccine dhp + l4 |
| Nobivacc DHPL 2nd vacc | vac Lepto lapsed |
| Nobivacc DHPL booster | vac lepto only |
| Nobivace DHPL booster | vac puppy 3rd + lepto4 |
| RSPCA Dog BV DHPPi/L | Vac puppy lepto only 1st+2nd |
| RSPCA Dog Prim V1 DHPPi/L | vac restart lepto 4 |
| RSPCA Dog Prim V2 DHPPi/L | vac top up L4 |
| top up lepto | Vacc 1yr Lepto |
| top up letpo | vacc 1yr lepto |
| vac puppy single | vacc 2w lepto |
| vac 2nd - restart | Vaccine L2 Batch number |
| vac 2nd breeder discount | Vaccine L4 Batch number |
| vac 2nd DHP+L4 | Vanguard 7 (25 box) |
| Vac 2nd Lepto (late) | Vanguard 7 (25) (per dose) |
| vac 2nd Lepto 4 restart | vanguard 7 2nd vacc |
| vac Adult dog restart DHP/Lepto4 | Vanguard 7 Vaccine |
| vac booster dog adult lepto 4 | Vanguard 7 Vaccine (25) |
| vac booster lepto 2 | Vanguard CPV-L |
| vac dog 1st | Vanguard CPV-L (25 box) |
| Vac dog Booster | Vanguard Cpv-L (25) |
| vac dog DHP/L4 | Vanguard Lepto |
| vac dog OOD | Vanguard Lepto C.I. (25) |
| vac dog ood booster | Vanguard Lepto CI (25 box) |
| vac error (only lepto 4 done) | Vanguard Lepto Ci (25) (per dose) |
| vac L4 | Versican + L4 |
| vac lapsed dog (L4) - prepaid | versican DHPPiL |
| vac lepto | Versican Plus DHPPI + L4 vaccine (25) |
| vac lepto 2nd | versican plus l4 |
| vac lepto 4 |  |

**
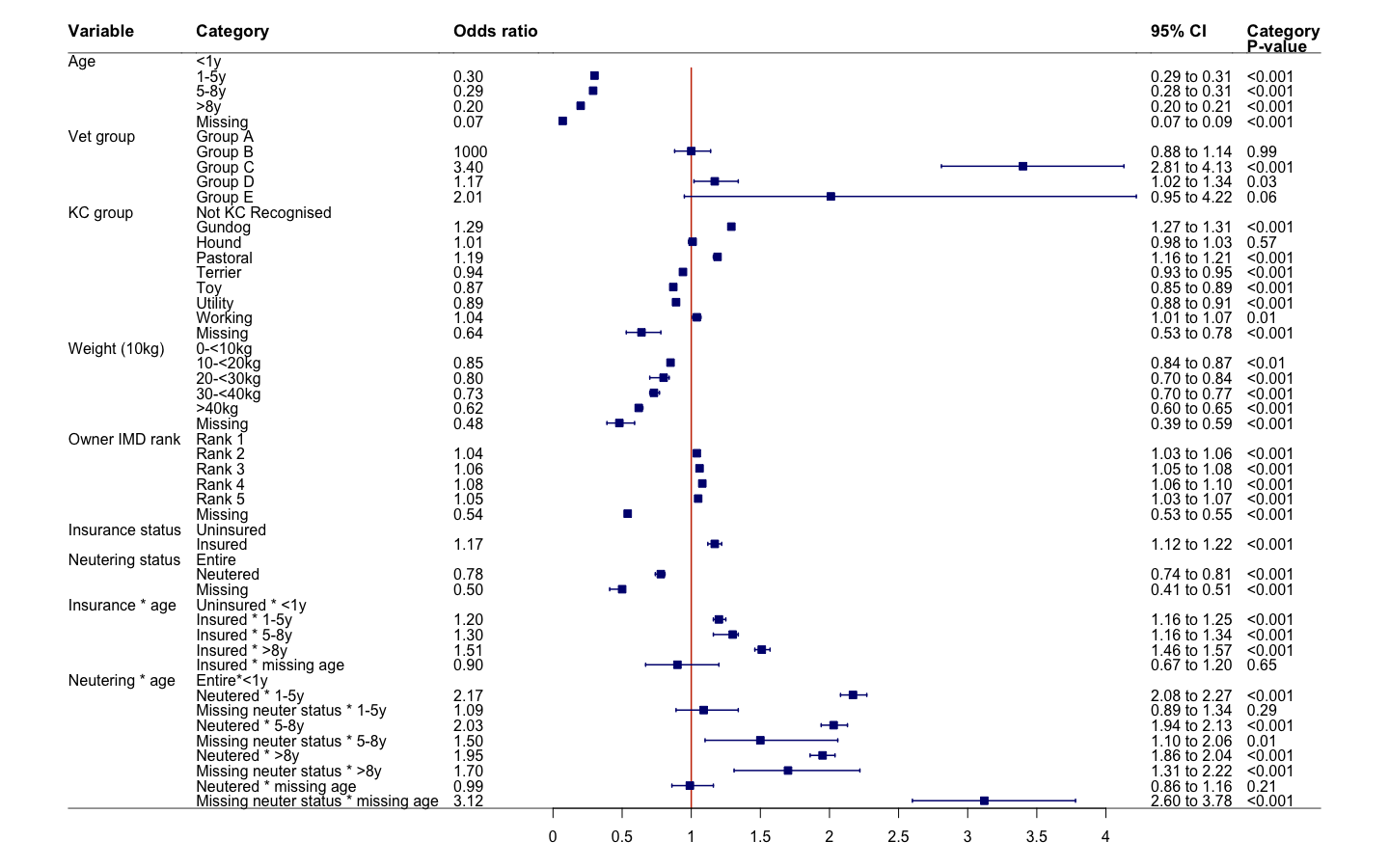
**

**Figure S1- Results of a second mixed effects model examining significant variables associated with leptospirosis vaccination administration for dogs under primary veterinary care in 2016.** The final model included neutering status, insurance status, Kennel Club group, bodyweight (10kg intervals), owner IMD rank, age and corporate group. Interaction terms between age and neutering and age and insurance are shown. Missing data was retained and is labelled ‘missing’ here.
